# 

**Published:** 1898-11-26

**Authors:** 


					THE HOSPITAL. "The standard of highest purity."?Lancet. Nov. 26th, 1898. A
. CADBURY'S pf* ?f CADBURY'S
OOOOA 1 ML  OOCOA
ABSOLUTELY PURE, NO DRUGS OR ALKALIES USED.
iw wv c??d
i\n Roe TT .
??? 635. Vol. ?X7.~gKS5?l Satuedat,
Nov. 26th, 1898.
iivju.um-LAMa.jiun rr/Ln jv
[BubsoriptionTerms,] pBICE TWOPENCE.

				

